# Annual acknowledgement of manuscript reviewers

**DOI:** 10.1186/1472-6947-13-74

**Published:** 2013-08-14

**Authors:** Adrian Aldcroft

**Affiliations:** 1 BioMed Central, Floor 6, 236 Gray’s Inn Road, London WC1X 8HB, UK

## Abstract

**Contributing reviewers:**

The editors of *BMC Medical Informatics and Decision Making* would like to thank all our reviewers who have contributed to the journal in Volume 12 (2012).

## 

Jos Aarts

Netherlands

Wilco Achterberg

Netherlands

Bram Adams

Canada

Samantha Adams

Netherlands

Rocco Agostino

Italy

Amir Ahmad

India

John Ainsworth

United Kingdom

Grace Ajuwon

Nigeria

Aamir Jalal Al Mosawi

Iraq

Laurence Alpay

Netherlands

K. Rivet Amico

USA

Ashwin Ananthakrishnan

USA

Milena Anatchkova

USA

Lefteris Angelis

Greece

Carmel Apicella

Australia

Joan Ash

USA

Caroline Asiimwe

Uganda

Parisa Aslani

Australia

Koray Atalag

New Zealand

Alexander Avidan

Israel

Wassim Ayadi

Tunisia

Franz Babl

Australia

Heather Baer

USA

James Baggs

USA

James Bailey

USA

Ubirajara Barroso Jr.

Brazil

Diana Bartlett

USA

Melissa Baysari

Australia

Michael Becich

USA

Carol Bennett

Canada

Adrian Benton

USA

Ariel Beresniak

Switzerland

Francois Berthoux

France

Jinbo Bi

USA

Chunhua Bian

China

Carlo Bianca

Italy

Jesus Bisbal

Spain

Seiji Bito

Japan

Mitchel Blair

United Kingdom

Ingrid Böhm

Germany

Stefano Bonacina

Italy

Albert Boonstra

Netherlands

Renata Bortolus

Italy

Rj Bosman

Netherlands

Hayden Bosworth

USA

Matt-Mouley Bouamrane

United Kingdom

Andrew Bradley

Australia

Elizabeth Bradley

USA

John F P Bridges

USA

Matthew Bultitude

United Kingdom

Howard Burkom

USA

Aidan Byrne

United Kingdom

Alejandro Calderon

Spain

Carlos Cano Gutierrez

Spain

María Del Carmen Carnero

Spain

Joao Paulo Carvalho

Portugal

Kate Cavanagh

United Kingdom

Sandeep Chandana

USA

Rui Chang

USA

Prodromos Chatzoglou

Greece

Rosa Chaves

Spain

Austin Chen

Taiwan

Kewei Chen

USA

Yu-Chun Chen

Taiwan

Carol Chenoweth

USA

Zaida Chinchilla-Rodriguez

Spain

Jen-Hsiang Chuang

Taiwan

Edward Cokely

USA

Nananda Col

United Kingdom

Jamie Coleman

United Kingdom

Isabelle Colombet

France

Kirsten Colpaert

Belgium

Jose Conde

Puerto Rico

Craig Conover

USA

Andrew Cook

United Kingdom

Jeremy Cooperstock

Canada

Alasdair Corfield

United Kingdom

Carlos Costa

Portugal

Kathrin Cresswell

United Kingdom

Jesse Crosson

USA

Rebecca Crowley

USA

Ricardo Cruz-Correia

Portugal

Elizabeth Cummings

Australia

Helen Dakin

United Kingdom

Jiangbo Dang

USA

Stefan Darmoni

France

Andrew Davidson

Australia

Gabrielle Davie

New Zealand

Nicolette De Keizer

Netherlands

Fabrizio De Ponti

Italy

Anke De Veer

Netherlands

Guilherme Del Fiol

USA

Nico Dellaert

Netherlands

Brenda Den Oudsten

Netherlands

Marie-Dominique Devignes

France

Joseph Diaz

USA

Brian Dixon

USA

Radu Dobrescu

Romania

James Dolan

USA

Wen Dong

USA

Shyamala Doraisamy

Malaysia

Charalampos Doukas

Greece

Jenny Doust

Australia

Dawn Dowding

United Kingdom

Tony Dowell

New Zealand

Matthew D’Souza

Australia

Georg Duftschmid

Austria

Claudio Eccher

Italy

Stamatis Efstathiou

Greece

Joakim Ekberg

Sweden

Arthur Elstein

USA

William Encinosa

USA

Saeid Eslami

Netherlands

Themis Exarchos

Greece

Julio Facelli

USA

Christopher Farmer

United Kingdom

Karen Farris

USA

Bernard Fernando

United Kingdom

Oscar Ferrandez

USA

Antonino Fiannaca

Italy

Carol E Fletcher

USA

Eric Ford

USA

Hamish Fraser

USA

Clark Freifeld

USA

Christoph M. Friedrich

Germany

Marie-Pierre Gagnon

Canada

Robert Gagnon

Canada

Bill Galanter

USA

Miguel Garcia-Perez

Spain

Rocio Garcia-Retamero

Spain

Sebastian Garde

Germany

Amit Garg

Canada

Teresa Gavaruzzi

United Kingdom

Colin Geddes

United Kingdom

Andrew Georgiou

Australia

Karthik Ghosh

USA

Julie Glanville

United Kingdom

John Glaser

USA

Nancy Glass

USA

Julien Gobeill

France

Mireille Goetghebeur

Canada

Ira Goldstein

USA

Shaun Grannis

USA

Kathleen Gray

Australia

Jeffrey Green

USA

Patricia Grocott

United Kingdom

Martha Grootenhuis

Netherlands

Francesca Guerriero

Italy

Arif Gulten

Turkey

Jose Gutierrez-Maldonado

Spain

Stuart Haines

USA

Patricia Halfon

Switzerland

W. Edward Hammond

USA

David Hanauer

USA

Priscilla Harries

United Kingdom

Mohamed Azmi Hassali

Malaysia

Gillian Hayes

USA

Emma Heeley

Australia

Annemaeri Hellebek

Denmark

Vitaly Herasevich

USA

Kurt E Hersberger

Switzerland

Peter Heywood

Australia

Thomas Higgins

USA

Oliver Hirsch

Germany

Valeria Hirschler

Argentina

Anne Holbrook

Canada

Richard Holden

USA

Matt Holford

USA

Inger Holmstrom

Sweden

Carol Homko

USA

Michael Horberg

USA

Alexander Horsch

Germany

Jan Horsky

USA

Monica Horvath

USA

Chien-Chin Hsu

Taiwan

Jiin-Chyr Hsu

Taiwan

Rebecca Hubbard

USA

Ursula Hübner

Germany

John Hurdle

USA

Samantha Hurst

USA

Abdulzahra Hussain

United Kingdom

Jan Illing

United Kingdom

David Isern

Spain

William Jacot

France

Arsh Jain

Canada

Monique Jaspers

Netherlands

Ian Jenkins

USA

Ane Johannessen

Norway

Kay Jones

Australia

Ray Jones

United Kingdom

Kristin Kahle-Wrobleski

USA

Ananth Kalyanaraman

USA

Bridget Kane

Ireland

Hyun-Sik Kang

Korea, South

Kyuchang Kang

Korea, South

Murat Kantarcioglu

USA

Sarvnaz Karimi

Australia

Dimitris Karlis

Greece

Maria Katapodi

USA

Zalman Kaufman

Israel

G Kim

USA

Ann Marie Kimball

USA

Patricia Kipnis

USA

Ela Klecun

United Kingdom

Caprice Knapp

USA

Petra Knaup

Germany

Sabine Koch

Sweden

Andrzej Kochut

USA

Marita Koivunen

Finland

Yiannis Kompatsiaris

Greece

Ross Koppel

USA

Ivica Kopriva

Croatia

Blanka Koristkova

Czech Republic

Ioannis Korkontzelos

United Kingdom

Jude Kornelsen

Canada

Irene Korstjens

Netherlands

Sotiris Kotsiantis

Greece

Vessela Krasteva

Bulgaria

Anand Krishnan

India

Jennifer Kryworuchko

Canada

Sharon Kuhlmann

Sweden

Alex Kuo

Canada

Mila Kwiatkowska

Canada

Poh-Chin Lai

Hong Kong

Margareta Larsson

Switzerland

Marek Laskowski

Canada

Annie Lau

Australia

Francis Lau

Canada

Kay Lawton

USA

Annie Leblanc

USA

Peter Leijdekkers

Australia

Heinz Lemke

Germany

Carlos León De Mora

Spain

Jacques Lepercq

France

Stephen J Leslie

United Kingdom

Guangxi Li

USA

Jingshan Li

USA

Jiuyong Li

Australia

Siaw-Teng Liaw

Australia

Carlos Lima

Portugal

Kevin Livingston

USA

William Long

USA

Martin Lopez-Nores

Spain

Anália Lourenço

Portugal

Pietro Lovaglio

Italy

Adolfo Lozano-Tello

Spain

Joanne Luciano

USA

Jon Lurie

USA

Marie Louise Luttik

Netherlands

Huifang Ma

China

Michael Maaser

Germany

Thusitha Mabotuwana

New Zealand

Velio Macellari

Italy

Charles Maclean

USA

Abdulraheem Mahmoud

Nigeria

Bradley Malin

USA

Victor Maojo

Spain

Maurice Mars

South Africa

Roger Marshall

New Zealand

Joel Martin

Canada

Linda Gore Martin

USA

Tsuji Masatsugu

Japan

Michael Matheny

USA

P S Mathuranath

India

Saverio Maviglia

United Kingdom

Ann Mcalearney

USA

Paul Mccullagh

United Kingdom

Clement Mcdonald

USA

Carolyn Mcgregor

Canada

Colleen Mchorney

USA

Molly Mcnett

USA

Sally Merry

New Zealand

Stephane Meystre

USA

Patrik Midlöv

Sweden

Ellen M Mikkelsen

Denmark

Armin Mikler

USA

Laura Militello

USA

Anne Miller

USA

Deborah Miller

USA

Anne Moen

Norway

Stefanie Monod

Switzerland

Luiz Monteiro

Brazil

Andrew Mooney

United Kingdom

Rutu Mulkar-Mehta

USA

Elizabeth Murray

United Kingdom

Juha Mykkänen

Finland

Radhakrishnan Nagarajan

USA

Meredith Nahm

USA

Candace Nelson

USA

Aurelie Neveol

USA

Kwan Hoong Ng

Malaysia

Matthew Nielsen

USA

Julie Niès

France

Christian Nohr

Denmark

Marlies Noordzij

Netherlands

Beverley Norris

United Kingdom

Pirkko Nykänen

Finland

Mikael Nystrom

Sweden

Olumuyiwa Odusanya

Nigeria

Brenda O’Neill

United Kingdom

Stephen O’Neill

United Kingdom

Isaac Opole

USA

Dermot O’Reilly

United Kingdom

Neli Ortega

Brazil

Juan Ospina

Colombia

Timothy Page

USA

Alan Palmer

United Kingdom

Supasit Pannarunothai

Thailand

George Panoutsos

United Kingdom

José Páramo

Spain

Jae-Hyeung Park

Korea, South

Philip Payne

USA

Alejandro Pazos

Spain

Lars Henning Pedersen

Denmark

Niels Peek

Netherlands

David Peiris

Australia

Yonghong Peng

United Kingdom

Beatriz Pérez

Spain

Goran Petersson

Sweden

Eduardo Pinheiro

Portugal

Habibollah Pirnejad

Netherlands

Klaus Pommerening

Germany

Eric Poon

USA

Cristian Pop-Eleches

USA

Mihail Popescu

USA

Kevin Pottie

Canada

John Powell

United Kingdom

Kamal Premaratne

USA

Catherine Quantin

France

Alexander Quarshie

USA

Gwenole Quellec

France

Brian Quilliam

USA

Heri Ramampiaro

Norway

Rebecca Randell

United Kingdom

Dimitri Aristotle Raptis

Switzerland

Debra Revere

USA

Stephen Rice

USA

Peter Richardson

USA

Michael Rieder

Canada

Patricia Riley

USA

Ian Ring

Australia

Colin Robertson

Canada

Jean Marie Rodrigues

France

Younghoon Roh

Korea, South

Sarah Ross

United Kingdom

Pietro Rubegni

Italy

Paul Rubel

France

Pekka Ruotsalainen

Finland

Alissa Russ

USA

Shelly Sachdeva

India

Charles Safran

USA

Krishna Saha

USA

G.P. Samanta

India

Sandhya Samarasinghe

New Zealand

Masaki Samejima

Japan

Peter Schattner

Australia

Gordon Schiff

USA

Christos Schizas

Cyprus

Martijn Schuemie

Netherlands

Jochen Schuld

Germany

Matthew Scotch

USA

Hanna Seidling

Germany

Pamela Semanik

USA

Karen Sepucha

USA

Enbal Shacham

USA

Tatyana Shamliyan

USA

Lawrence Sherman

USA

Catherine Sherrington

Australia

Kit Simpson

USA

Dean Sittig

USA

Ruth Sladek

Australia

Daniel Slamanig

Austria

Neil Smalheiser

USA

Catherine Smith

USA

Soegijardjo Soegijoko

Indonesia

Hernan Solari

Armenia

Min Song

Korea, South

Daby Sow

USA

Irena Spasic

United Kingdom

Stuart Speedie

USA

James Stahl

USA

Melissa Stockwell

USA

Lacramioara Stoicu-Tivadar

Romania

Sharon Straus

Canada

Fengxi Su

China

Sean Sullivan

USA

Tan-Hsu Tan

Taiwan

Luis Tari

USA

Reiji Teramoto

Japan

Carl Thompson

United Kingdom

Lau Thygesen

Denmark

Patrick Tighe

USA

Myriam Torres

USA

Elizabeth Townsend

Canada

Andrea Tricco

Canada

Marc Triola

USA

Vasilis Tsimihodimos

Greece

Artemis Tsitsika

Greece

Aris Tzavaras

Greece

Shahadat Uddin

Australia

Yasunori Utsunomiya

Japan

Faith-Michael Uzoka

Canada

Mojtaba Vaismoradi

Iran

Jenny Theodora Van Der Steen

Netherlands

Th P P Van Der Weide

Netherlands

Erik Van Eaton

USA

Trevor Van Mielro

Canada

Mark Van Oyen

USA

Cornelia Van Uden-Kraan

Netherlands

Jodi Vanden Eng

USA

Martijn Vastenburg

Netherlands

Arvind Venkat

USA

Herna Viktor

Canada

Rafael Villanueva

Spain

Vivian Vimarlund

Sweden

Stephen Vindigni

USA

Robert Volk

USA

Henrik Von Wehrden

Germany

Stanimir Vuk-Pavlovic

USA

Levi Waldron

USA

Byron Wallace

USA

Jacco Wallinga

Netherlands

Aaron Ward

Canada

Jim Warren

New Zealand

Kaaren Watts

Australia

Richard Weisler

USA

Kuang-Yi Wen

USA

Chunhua Weng

USA

Josh West

USA

Joseph Wherton

United Kingdom

Laura White

USA

Robyn Whittaker

New Zealand

Margaret Williamson

Australia

Ann-Britt Wiréhn

Sweden

Klaus-Hendrik Wolf

Germany

William Woodall

USA

Deborah Wright

New Zealand

Robert Wu

Canada

Xiaogang Wu

USA

Rolf Wynn

Norway

Fuzhong Xue

China

Minoru Yamada

Japan

Qing Yan

USA

Lin Yang

Hong Kong

Wen Yao

USA

Hal Yee

USA

Illhoi Yoo

USA

Reza Yousefi-Nooraie

Canada

Ping Yu

Australia

Emanuela Zanelli

Italy

Christina Zarcadoolas

USA

Steven Zeliadt

USA

Shaoting Zhang

USA

Wenle Zhao

USA

Maria Zolfo

Belgium

Stefano Zucchini

Italy

## Pre-publication history

The pre-publication history for this paper can be accessed here:

http://www.biomedcentral.com/1472-6947/13/74/prepub

